# Endometriosis foci differentiation by rapid lipid profiling using tissue spray ionization and high resolution mass spectrometry

**DOI:** 10.1038/s41598-017-02708-x

**Published:** 2017-05-31

**Authors:** Vitaliy V. Chagovets, Zhihao Wang, Alexey S. Kononikhin, Natalia L. Starodubtseva, Anna Borisova, Dinara Salimova, Igor A. Popov, Andrey V. Kozachenko, Konstantin Chingin, Huanwen Chen, Vladimir E. Frankevich, Leila V. Adamyan, Gennady T. Sukhikh

**Affiliations:** 1 V.I. Kulakov Research Center for Obstetrics, Gynecology and Perinatology, Ministry of Healthcare of the Russian Federation, 4 Oparina str., 117997 Moscow, Russia; 2 Jiangxi Key Laboratory for Mass Spectrometry and Instrumentation, East China University of Technology, 418 Guanglan road, 330013 Nanchang, China; 3 Moscow Institute of Physics and Technology, 141700 Dolgoprudnyi, Moscow Region Russia

## Abstract

Obtaining fast screening information on molecular composition of a tissue sample is of great importance for a disease biomarkers search and for online surgery control. In this study, high resolution mass spectrometry analysis of eutopic and ectopic endometrium tissues (90 samples) is done using direct tissue spray mass spectrometry in both positive and negative ion modes. The most abundant peaks in the both ion modes are those corresponding to lipids. Species of three lipid classes are observed, phosphatidylcholines (PC), sphingomyelins (SM) and phosphoethanolamines (PE). Direct tissue analysis gives mainly information on PC and SM lipids (29 species) in positive ion mode and PC, SM and PE lipids (50 species) in negative ion mode which gives complementary data for endometriosis foci differentiation. The biggest differences were found for phospholipids with polyunsaturated acyls and alkils. Although, tissue spray shows itself as appropriate tool for tissue investigation, caution should be paid to the interpretation of mass spectra because of their higher complexity with more possible adducts formation and multiple interferences must be taken into account. The present work extends the application of direct tissue analysis for the rapid differentiation between endometriotic tissues of different foci.

## Introduction

Endometriosis is an abundant gynecological pathology of poorly understood pathogenesis affecting 10% of women^1^. It is characterized by the extrauterine presence of endometrial glands and stroma. The disease affects mostly women in their reproductive age and can cause wide set of non-specific symptoms including infertility, dysmenorrhea, dyspareunia, and non-cyclic pelvic pain. The only reliable way to diagnose the pathology is surgical laparoscopy for the moment. Many efforts have been applied to find biomarkers of endometriosis and to develop less invasive methods to reveal the presence of early stages^2^. Such investigations were mainly devoted to body fluids screening^3–7^. Lack of the information about molecular composition of eutopic (inside uterine) and ectopic (extrauterine) endometrium is observed. There are hypotheses about alteration in composition of ectopic and eutopic endometrial tissues and in surrounding tissue which allows eutopic endometrium survival. Therefore, tissue investigation can shed some light on mechanisms of the disease and be used for validation of biomarkers found in fluids in low-invasive way. Data on eutopic and ectopic endometrium composition is also important for the development of the online surgery control to determine volume of an operation. This necessitates development of method for fast screening of big amount of tissue samples. Wide variety of approaches for tissue composition analysis are present nowadays^8^: matrix-assisted laser desorption/ionization imaging, desorption electrospray ionization, etc. One of the ambient methods for a sample analysis with minimal pretreatment is suggested by Cooks’ group and named “leaf-spray”^9^. This method was further extrapolated for fast tissue analysis and turned into different variations of tissue spray^10–15^. The method has been applied to the analysis of brain tissues and various cancer tumors^10, 13^.

The present work extends the application of direct tissue analysis for the rapid differentiation between endometriotic tissues of different foci. Features of different tissues are identified by conventional lipidomic approach including lipid extraction and following analysis by hydrophilic interaction liquid chromatography with electrospray ionization mass spectrometry (HILIC-LC/ESI-MS)^16, 17^.

## Results

**Figure 1:**
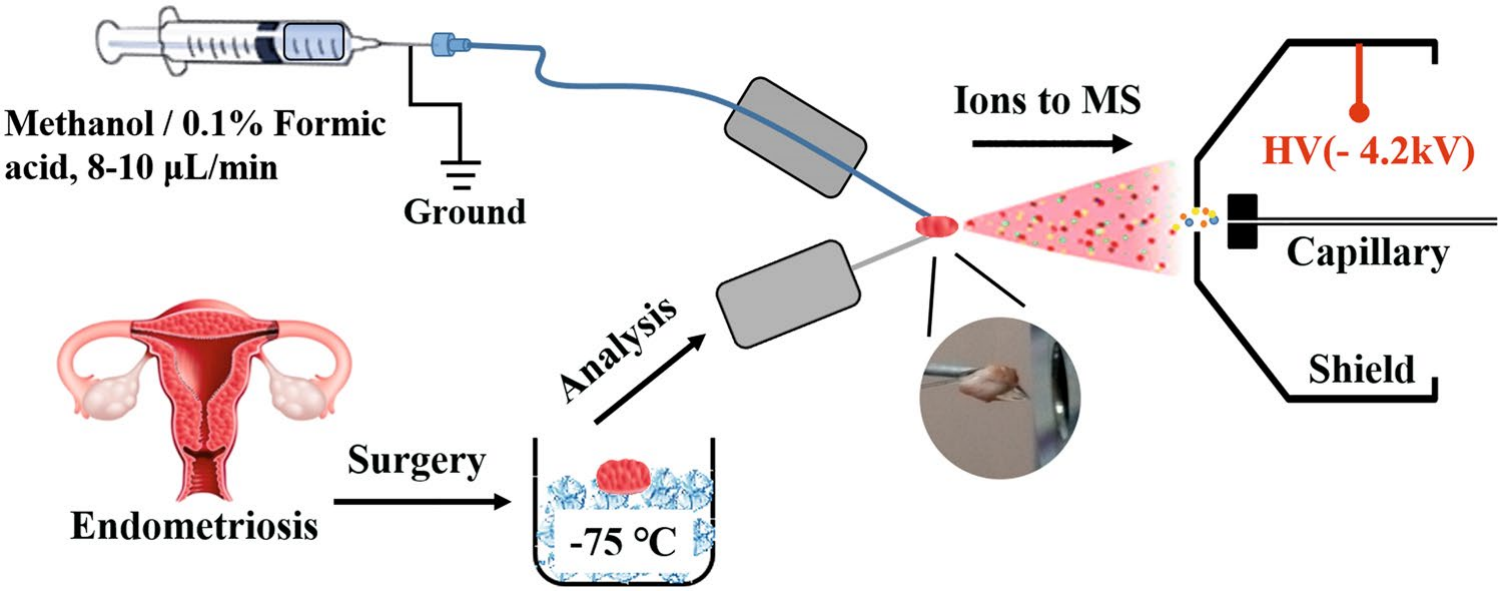
Flowchart of the experiment and schema of the direct tissue spray ion source.

The design of MS analysis of tissue samples and the ion source scheme are presented in Fig. 1. The following settings are varied until obtaining stable TIC in both positive and negative ion modes: distance from tissue tip to the mass spectrometer inlet is varied in the range 5–50 mm; applied potential in the range 2–5 kV, extracting solvent flow rate in the range 5–50 µL/min. Figure S1 shows extracted positive ion chromatograms of some ions obtained with optimized settings. Relative standard deviation of a peak intensity is within 5% for the same tissue piece and about 5–10% for neighboring pieces. Then the scheme of an analysis for every tissue sample is as follows. A piece of tissue of approximate size 2 × 1 × 1 mm is cut from a frozen sample, thawed and fixed on a needle. After that, an MS analysis starts. The suggested construction gives better tissue fixation and control.

### Positive ion mode

**Figure 2:**
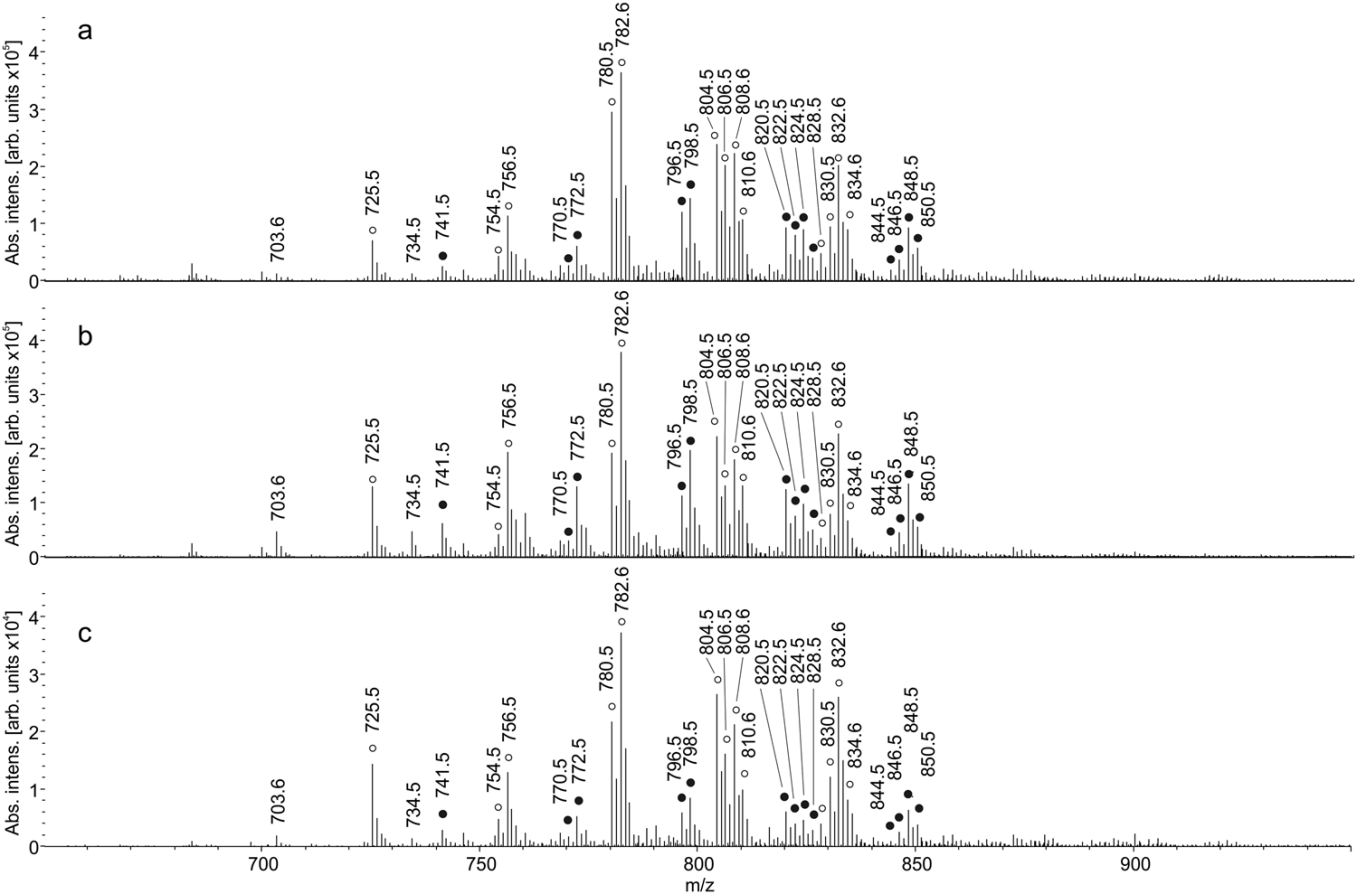
Positive ion tissue spray mass spectra of (**a**) eutopic endometrium; (**b**) endometriotic ovarian cyst; (**c**) pelvic endometriosis.

Representative mass spectra of three kinds of endometriotic tissue from one patient collected in positive ion mode are shown in Fig. 2. A total of 1159 ions in sample tissues in positive ion mode are detected over threshold of 200 counts in different tissue types. The most abundant m/z correspond to lipid species. The identification is done in the following way illustrated for *m/z* 782.5685. An accurate mass of a compound which abundance exceeds a threshold in 200 counts is found from the mass spectrum. Tentative assignment of the compound is done based on LIPID MAPS data^18^ within 10 ppm mass accuracy. For the considered m/z it can be either protonated species of PC 36:4, PE 39:4 or sodiated species of PC 34:1, PE 37:1. MS/MS information about the fragmentation pattern of the considered *m/z* is used for better assignment (Figure S2a). Peaks with *m/z* 147 and 184 are observed in the tandem mass spectra. These peaks are characteristic for fragmentation of sodiated and protonated phosphatidylcholine, correspondingly, and originate from its polar head group^19, 20^. This fact leads to a conclusion that *m/z* 782 precursor ion corresponds to interfered protonated PC 36:4 and sodiated PC 34:1. Such peak overlapping is known problem upon lipids study in positive ion mode^19, 21, 22^. Identified lipid species are listed in Table 1 and some of them are marked with opened circles in Fig. 2. Additional lipids validation is done by HILIC-LC/MS analysis of tissue’s lipid extract using methods described earlier^16, 17^. Figure S3 represents resulting TIC from HILIC-LC/MS analysis of the lipid extract. Annotated chromatographic peaks correspond to lipid species observed in tissue spray experiments. Retention time of the observed lipids (Table S2) correlates with literature data^16, 17^ and confirms identification provided in Tables 1 and 2.

**Table 1:** Theoretical and experimental *m/z* values of lipid species identified in positive ion mode in tissue samples with measured mass accuracy.

Lipid	[M + Na]^+^			[M + O + Na]^+^		
	Theoretical	Experimen- tal	Mass accuracy [ppm]	Theoretical	Experimen- tal	Mass accuracy [ppm]
**Phosphatidylcholines**						
PC 30:0	728.5200	728.5237	5	744.5160	n.d.	
PC O-32:0	742.5721	742.5665	8	758.5635	n.d.	
PC 32:1	754.5357	754.5393	5	770.5308	770.5278	4
PC 32:0	756.5513	756.5537	3	772.5476	772.5480	1
PC O-34:1	768.5877	768.5824	7	784.5805	784.5846	5
PC 34:2	780.5513	780.5544	4	796.5467	796.5436	4
PC 34:1	782.5670	782.5700	4	798.5624	798.5656	4
PC O-36:4	790.5721	790.5651	9	806.5647	806.5630	2
PC 36:4	804.5513	804.5543	4	820.5462	820.5444	2
PC 36:3	806.5670	806.5606	8	822.5456	822.5549	11
PC 36:2	808.5826	808.5856	4	824.5771	824.5736	4
PC 36:1	810.5983	810.6011	3	826.5911	826.5878	4
PC O-38:5	816.5877	816.5893	2	832.5814	832.5909	11
PC 38:7	826.5357	826.5368	1	842.5280	842.5401	14
PC 38:5	830.5670	830.5700	4	846.5605	846.5595	1
PC 38:4	832.5826	832.5859	4	848.5777	848.5749	3
PC 38:3	834.5983	834.5958	3	850.5932	850.5853	9
PC 40:7	854.5670	854.5654	2	870.5591	870.5661	8
PC 40:6	856.5826	856.5858	4	872.5739	872.5719	2
**Sphingomyelins**						
SM 32:1	697.5254	697.5318	9	713.5203	713.5264	9
SM 33:1	711.5411	711.5477	9	727.5360	727.539	4
SM 34:2	723.5411	723.5477	9	739.5360	739.5421	8
SM 34:1	725.5567	725.5628	8	741.5516	741.5539	3
SM 36:1	753.5880	753.5934	7	769.5829	769.5848	2
SM 38:1	781.6193	781.6154	5	797.6142	797.6107	4
SM 40:1	809.6506	809.6539	4	825.6455	825.6507	6
SM 42:3	833.6506	833.6535	3	849.6455	849.6479	3
SM 42:2	835.6663	835.6737	9	851.6612	851.6645	4
SM 42:1	837.6819	837.6860	5	853.6768	853.6757	1

**Table 2:** Theoretical and experimental *m/z* values of lipid species identified in negative ion mode in tissue samples with measured mass accuracy.

Lipid	Theoretical	Experimental	Mass accuracy [ppm]
**Phosphatidyletanoamines [M − H]** ^**−**^			
PE O-34:2	700.5287	700.5357	10
PE 34:1	716.5236	716.5302	9
PE O-36:5	722.5130	722.5198	9
PE O-36:3	726.5443	726.5465	3
PE O-36:2	728.5599	728.5622	3
PE 36:4	738.5079	738.5062	2
PE 36:2	742.5392	742.5417	3
PE 36:1	744.5549	744.5569	3
PE O-38:7	746.5130	746.5145	2
PE O-38:6	748.5286	748.5323	5
PE O-38:5	750.5443	750.5491	6
PE 38:7	760.4922	760.4952	4
PE 38:6	762.5079	762.5052	4
PE 38:5	764.5235	764.5308	9
PE 38:4	766.5392	766.5364	4
PE O-40:6	776.5599	776.5565	4
PE O-40:5	778.5756	778.5728	4
PE 40:8	786.5079	786.5156	10
PE 40:7	788.5235	788.5256	3
PE 40:5	792.5548	792.5510	5
PE 40:4	794.5705	794.5743	5
PE 42:10	810.5079	810.5096	2
PE 42:9	812.5235	812.5269	4
**Phosphatidylcholines [M + Cl]** ^**−**^			
PC 30:0	740.50018	740.5013	2
PC O-32:0	754.55228	754.5545	3
PC 32:1	766.51588	766.5214	7
PC 32:0	768.53148	768.5325	1
PC O-34:1	780.56788	780.5639	5
PC 34:2	792.53148	792.5296	2
PC 34:1	794.54718	794.545	3
PC O-36:4	802.55228	802.5551	4
PC 36:4	816.53148	816.5299	2
PC 36:3	818.54718	818.5456	2
PC 36:2	820.56278	820.5605	3
PC 36:1	822.57848	822.5703	10
PC O-38:5	828.56788	828.5644	4
PC 38:7	838.51588	838.5127	4
PC 38:5	842.54718	842.5397	9
PC 38:4	844.56278	844.5558	8
PC 38:3	846.57848	846.5731	6
PC 40:7	866.54718	866.5383	10
PC 40:6	868.56278	868.5582	5
**Sphingomyelins [M + Cl]** ^**−**^			
SM 32:1	709.50558	709.5011	6
SM 33:1	723.52128	723.5198	2
SM 34:2	735.52128	735.5283	10
SM 34:1	737.53688	737.5351	2
SM 36:1	765.56818	765.5732	7
SM 42:3	845.63078	845.6332	3
SM 42:2	847.64648	847.6545	9
SM 42:1	849.66208	849.6568	6

Series of peaks denoted with closed circles are worth of consideration (Fig. 2). Their profile is similar to that of the opened circle peaks. The difference between these two groups is 16 Da which can evidence about oxidation of lipids during direct tissue analysis. Such effect has been observed in another ambient method, DESI^23^. This conclusion is supported by the presence of *m/z* 163 peak in tandem mass spectra of *m/z* 799 (Figure S2b). It is also 16 Da bigger then the characteristic fragment of polar head group of sodiated PC. Another explanation of this series can be potassium cation attachment to the initial lipid molecules.

**Figure 3:**
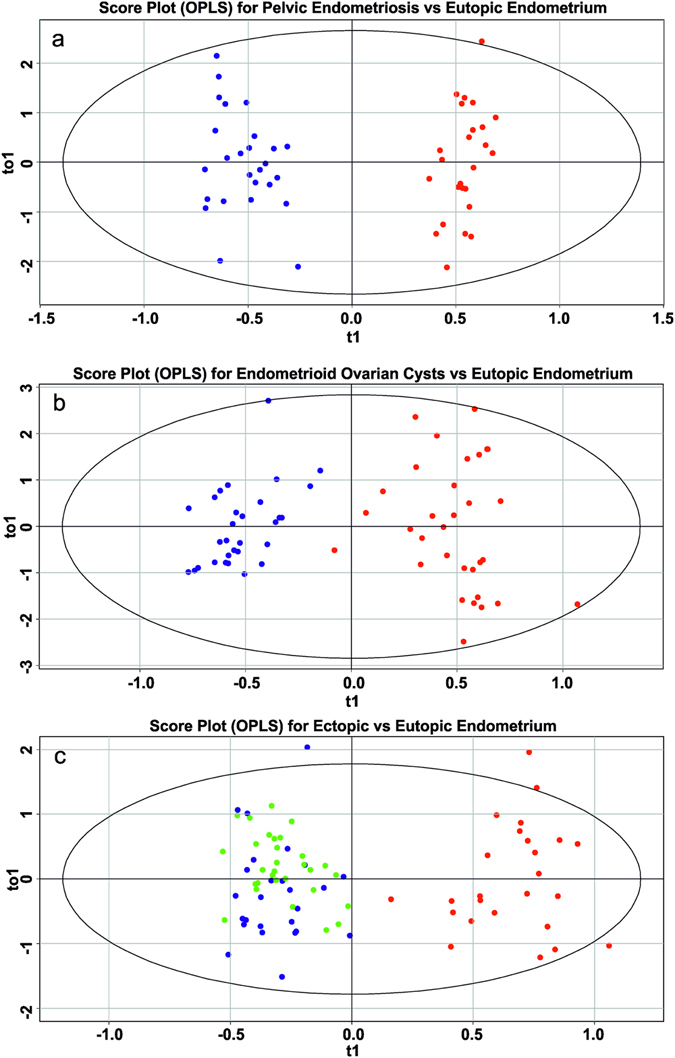
Score plots of multivariate data analysis of relative abundances of ions in positive ion mode using the OPLS-DA method (**a**) pelvic endometriosis *vs* eutopic endometrium; (**b**) endometriotic ovarian cysts *vs* eutopic endometrium; (**c**) ectopic *vs* eutopic endometrium.

The supervised OPLS-DA model is used to separate different tissue types and find the differentially produced metabolites. As shown in Fig. 3, the OPLS-DA model can separate eutopic endometrium from ectopic one, while two types of endometriotic foci are not clustered demonstrating higher similarity compared to eutopic endometrium (Fig. 3c). R^2^ values representing explained variance of the data are extracted from the models and listed in Table S3. Predictive capability of the models is estimated by Q^2^. This parameter is obtained with Leave-one-out cross-validation (LOOCV) and the resulting values are presented in Table S3. All models show a reasonable predictive ability.

### Negative ion mode

**Figure 4:**
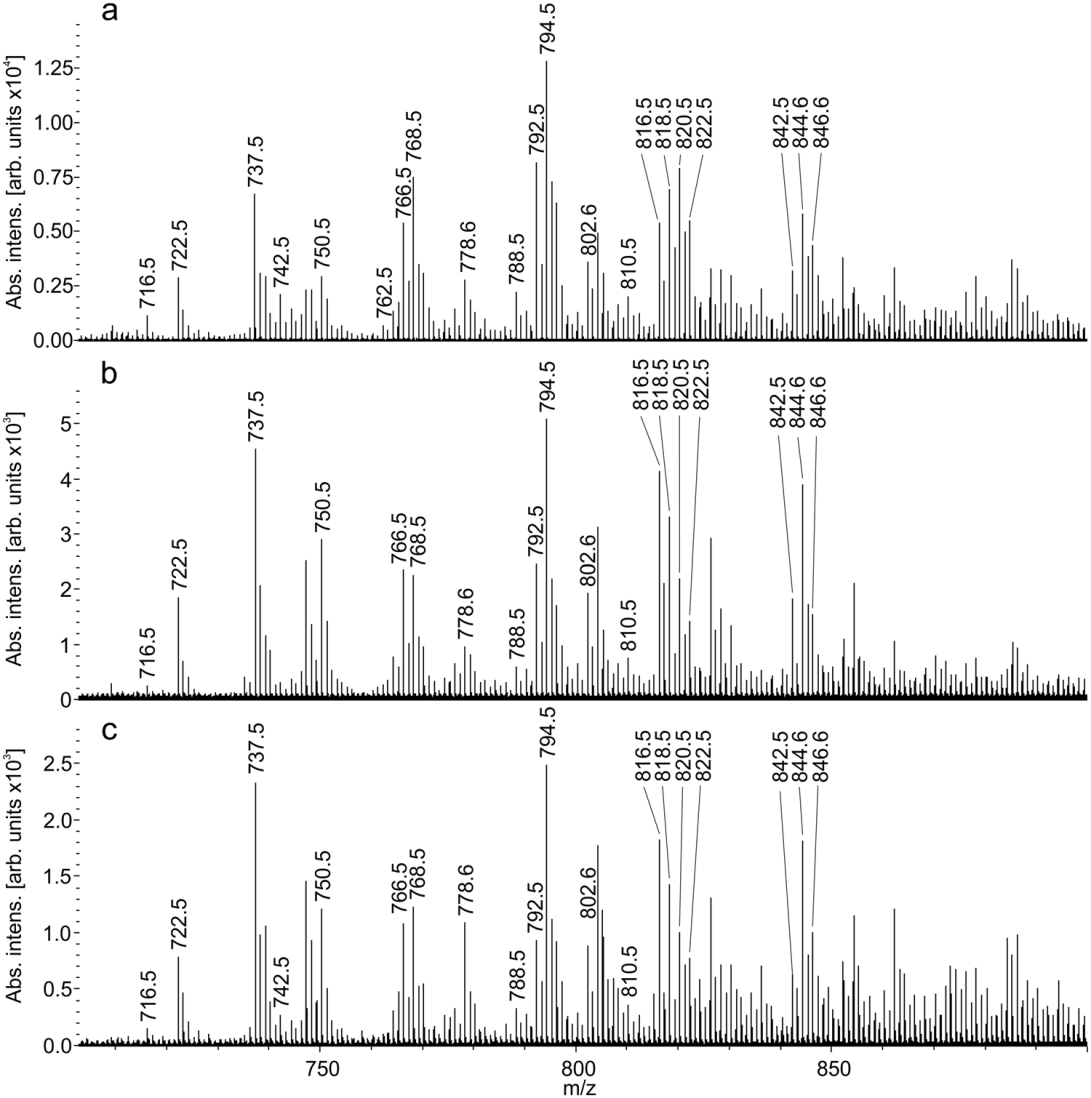
Negative-ion tissue spray mass spectra of (**a**) eutopic endometrium; (**b**) endometriotic ovarian cyst; (**c**) pelvic endometriosis.

Figure 4 demonstrates characteristic mass spectra of tissue samples in negative ion mode. 307 peaks in the mass range 400–1000 are detected over 20 counts threshold. Identification in negative ion mode is done in the same way as in the positive one. It is done according to accurate mass within 10 ppm and tandem mass spectra. The principal difference between tandem mass spectra of lipids in positive and negative ion modes is that in positive ion mode product ions which characteristic for lipid class are generated, while in negative ion mode the most probable products are those of fatty acids. So different algorithms for tandem mass spectra processing are used. The list of identified lipids is in the Table 2. Lipids of three classes are found in the mass spectra. Namely, phosphatidylcholines, sphingomyelins and ethanolamines. PC and SM are registered as adducts with chloride ion and PE are in the form of deprotonated molecules. It is worth noting that negative ion mode allows registration of the PE species in contrast to positive ion mode where they undergone signal suppression by choline-containing lipids.

**Figure 5:**
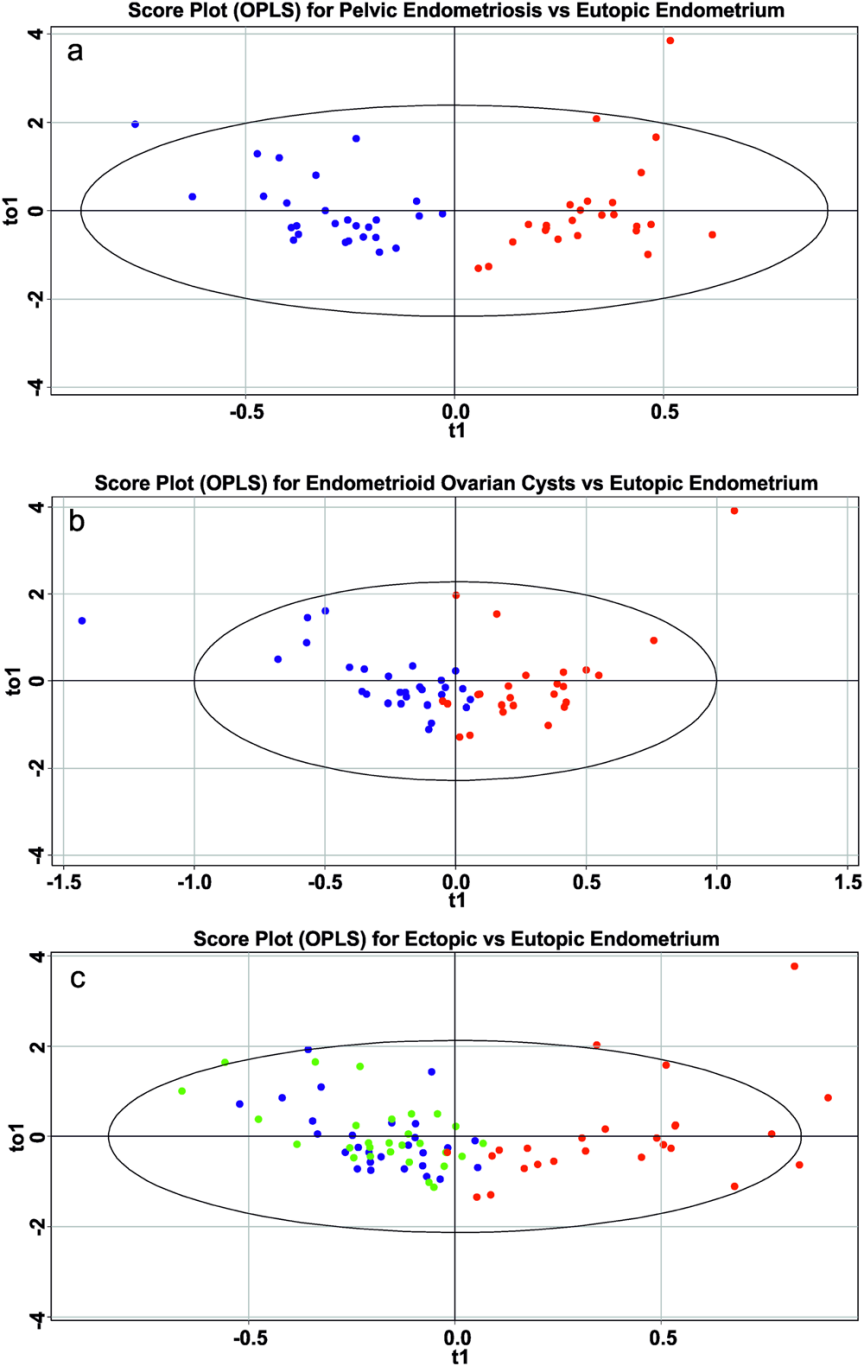
Score plots of multivariate data analysis of relative abundances of ions in negative ion mode using the OPLS-DA method (**a**) pelvic endometriosis *vs* eutopic endometrium; (**b**) endometriotic ovarian cysts *vs* eutopic endometrium; (**c**) ectopic *vs* eutopic endometrium.

The OPLS-DA score plots are shown in Fig. 5. Eutopic endometrium tissues are also clearly separated from ectopic but the models for negative ions are characterized by somewhat worth parameters (Table S4).

### Comaprative lipid profile analysis for endometriotic foci differentiation

**Figure 6:**
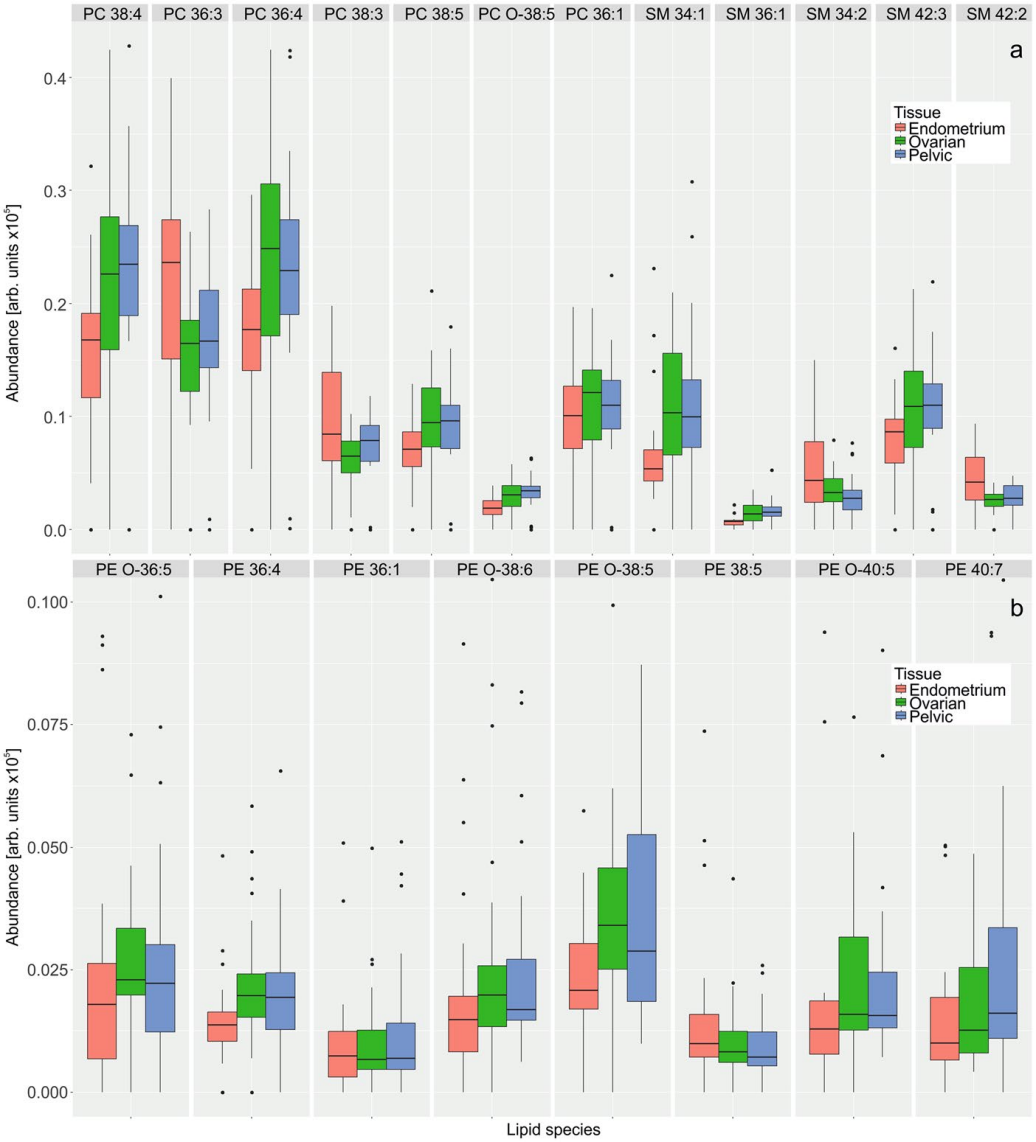
Comparison of relative abundances of lipid species with the highest VIP scores in normal and endometriotic tissues of thirty endometriosis patients determined using direct tissue analysis in (**a**) positive ion mode; (**b**) negative ion mode, only phosphatidylethanolamines are shown as they provide additional information in negative ion mode.

Variable influence on projection (VIP) is used in the study to find *m/z*’s the most contributed to the OPLS-DA models. The highest VIP values belong mainly to lipid species. The comparison box-and-whisker plots for these lipids are shown in Fig. 6. First 30 features with the highest VIP score are selected in positive ion mode data, and from that 30, 12 unique features identified as lipids are presented in the box-and-whisker plot (Fig. 6a). PC, PE and SM lipids are present among first 30 features with highest VIP according to negative ion data. Figure 6b demonstrates levels of the most significant PE measured in negative ion mode. Similar PC and SM species can be found among most significant features in both positive and negative ion mode but they are omitted in Fig. 6b to mark up additional information provided by PE which does not suffer from suppression effect from choline-containing compounds in negative ion mode. Abundances of most lipids are similar for ovarian and pelvic endometriosis and differ from eutopic endometrium.

Comparative study of normal and pathological tissues is a necessary step of biomarkers discovery and mechanisms of a disease development. Direct tissue analysis allows fast screening of a tissue composition. Mainly lipid constituents can be studied with such method if not to make some additional efforts. Among tissue differentiating compounds PC, SM and PE polyunsaturated lipids are identified. Species of these classes have been found as features of endometriosis in biofluids as well^3, 4, 7, 24^.

## Discussion

The feasability of endometriotic tissue type differentiation by tissue spray method is demonstrated. The differentiating features are several lipid species from PC, SM, PE classes. There are investigations of endometriosis based on lipid profiling but most of them use plasma, serum or peritoneal fluid as an object^4, 7, 24^. The same lipid classes were found to be altered in endometriosis patients compared to healthy ones. In the study of Bi-Cheng Yang with coworkers^7^, PC 38:4 and SM 34:1 are also among featured lipids as well as SM 34:2 in Vouk’s paper^24^. In Dutta’s work lipids from endometriosis mice serum and liver were profiled^4^. It was found that PEs were downregulated whereas SMs, PCs, lysoPCs, lysoPEs, and plasmeny-PEs were upregulated in endometriosis mice^4^. Observed lipids are essential participants of many important pathways. PC is one of the major sources of polyunsaturated fatty acids, which are the precursors of eicosanoids and has numerous biological activities. Abnormal PC metabolism is reported to exist in many disease^25, 26^. Sphingolipids are a distinctive and highly important class of lipids functioning in different biological processes such as signal transduction and cell fate determination^27^. Although the role of sphingolipids in endometriosis has not been fully investigated. Sphingolipids are increasingly known to be important bioactive signaling molecules and are involved in a diverse range of cellular processes^28^. PE is a substrate for numerous phospholipids of membrane cells, primarily phosphatidylcholine^29^. This may explain the phosphoethanolamine in endometrial tissues as phosphatidylethanolamine is a precursor of phosphatidylcholine^30^. Phosphoethanolamine is the most widespread lipid on the cytoplasmic membrane that is involved in different cellular activities, e.g. cell cycle, membrane fusion, autophagy and apoptosis^29^.

Although, tissue spray shows itself as appropriate tool for tissue investigation, caution should be paid to the interpretation of mass spectra because of their higher complexity with more possible adducts formation and multiple interferences must be taken into account. The biggest differences were found for phospholipids with polyunsaturated acyls and alkils. Direct tissue analysis gives mainly information on PC and SM lipids (29 species) in positive ion mode and PC, SM and PE lipids (50 species) in negative ion mode which gives complementary data for endometriotic foci differentiation.

## Methods

All experimental protocols are approved by the Ethical Committee of the V. Kulakov Research Center for Obstetrics, Gynecology and Perinatology (Moscow, Russia). All clinical investigations are conducted according to the principles expressed in the Declaration of Helsinki.

### Chemicals

Methanol, acetonitrile, 2-propanol, chloroform and formic acid of HPLC grade are purchased from Sigma-Aldrich (St. Louis, MO, USA). Deionized water is purchased from Panreac (Barcelona, Spain).

### Sample preparation

Endometrial tissues (90 samples) of different localization of 30 patients (Table S1) are obtained from the Department of Surgery, V. Kulakov Research Center for Obstetrics, Gynecology and Perinatology (Moscow, Russia). All patients have read and signed Informed consent approved by the Ethical Committee of the V. Kulakov Research Center for Obstetrics, Gynecology and Perinatology (Moscow, Russia). The classification of the American Fertility Society is used in this study^31^. All patients have stage III–IV of the outer genital endometriosis according to this classification. The mandatory criteria for inclusion of patients into the investigation are as follows: the reproductive age of women, the lack of hormonal and drug therapy for 6 months before surgery, the absence of inflammatory diseases of the pelvic organs and severe somatic pathology. The samples are frozen in liquid nitrogen immediately after surgery and stored under −75 °C until the analysis. For the analysis, a small piece of a sample (approximately 2 × 1 × 1 mm) is cut, thawed and fixed on the needle in the ion source (Fig. 1).

Lipid extracts are prepared according to a modified Folch method^32^. Briefly, 40 mg of tissue is homogenized in liquid nitrogen, 4 mL of chloroform–methanol (2:1, v/v) mixture is added to the sample and the mixture is incubated for 10 min. The homogenate is filtered using coarse filter paper. 800 µL of 1 mol/L NaCl is added, and the mixture is centrifuged at 3000 rpm for 5 min at the ambient temperature. The organic bottom layer containing lipids is evaporated with a stream of nitrogen and redissolved in acetonitrile-2-propanol (1:1, v/v) mixture for LC/MS analysis.

### Tissue spray conditions

MS analysis of tissue samples is performed using in-lab designed ion source (Fig. 1) similar to that described earlier^10^ but with minor modifications. Constant stream of H_2_O/methanol 1/9 is supplied to the tissue with flow rate of 1 mL/min by Dionex binary pump. Distance between a sample and MS inlet is about 5–10 mm. Applied potential between tissue and inlet capillary is 4.2 kV. Maxis Impact qTOF (BrukerDaltonics, Bremen, Germany) is used as a mass analyzer. The signal of a tissue is saved during 3 min after total ion current (TIC) equilibration. Analysis schedule is as follows: 3 minutes in positive ion mode, data dependent analysis (DDA) in positive ion mode, 3 minutes in negative ion mode, DDA in negative ion mode. Mass spectra are registered at a 2 Hz frequency resulting in 360 spectra for 3 minutes. The mass range is *m/z* 400–1000.

Tandem MS is done using DDA with the following characteristics. Three most abundant peaks are chosen after full mass scan and subjected MS/MS analysis with collision induced dissociation applying 35 eV collision energy, mass exclusion time is 1 minute.

### HILIC-LC conditions

Extract of endometriotic tissue is analyzed using a Spherisorb Si column (150 × 2.1 mm, 5 µm; Waters, Milford, MA, USA), a flow rate of 50 µL/min, an injection volume of 3 µL, column temperature of 40 °C and a mobile phase gradient: 0–0.5 min−6% B, 60.5 min−23% B, 61–64 min−6% B where solvent B is 5 mM aqueous ammonium acetate. Solvent A is acetonitrile^16, 17^. All LC/MS experiments are performed on the Dionex UltiMate 3000 liquid chromatograph (ThermoScientific, Germering, Germany) coupled to the Maxis Impact qTOF analyzer with ESI (Bruker Daltonics, Bremen, Germany).

### ESI-MS conditions

Maxis Impact qTOF is used in the HILIC-LC/MS method with ESI (Bruker Daltonics, Bremen, Germany). Mass spectra are obtained in positive ion and negative ion modes in the mass range m/z 400–1000 with the following setting of tuning parameters: capillary voltage 4.1 kV in positive ion mode (3.0 kV in negative ion mode), pressure of the nebulizing gas 0.7 bar, drying gas flow rate 6 L/min, and temperature of the drying gas 200 °C.

### Data analysis

Obtained mass spectra from each sample is averaged over 3 min and saved in *m/z* – Intensity tables using DataAnalysis software (BrukerDaltonica, Bremen, Germany). Thus obtained data is processed with scaling on TIC and peak alignment. Multivariate data analyses is performed using orthogonal projections onto latent structures discriminant analysis (OPLS-DA) method^33^ implemented in *ropls* package^34^. Multivariate models are described using R^2^ and Q^2^ parameters, where R^2^ describes fraction of data that the model can explain using the latent variables, and Q^2^ describes part of data predicted by the model according to the cross validation.

Tentative lipid identification is conducted with in-lab created R code. The code searches a record in LIPID MAPS database^18^ within 10 ppm from the experimental *m/z*. More precise identification is done based on the MS/MS data for the peak under consideration, if it undergone MS/MS analysis.

## Electronic supplementary material

Supplementary Information
