# Supplementary material for: Endometriosis foci differentiation by rapid lipid profiling using tissue spray ionization and high resolution mass spectrometry
Source: Sci Rep. 2017 May 31;7:2546. doi: 10.1038/s41598-017-02708-x (PMC5451410; doi:10.1038/s41598-017-02708-x)

**SUPPORTING INFORMATION**

**Endometriosis foci differentiation by rapid lipid profiling using tissue spray ionization and high resolution mass spectrometry**

Vitaliy Chagovets^1^; Zhihao Wang^1,2^; Alexey Kononikhin^1,3^; Natalia Starodubtseva^1,3^, Anna Borisova^1^; Dinara Salimova^1^, Igor Popov^1,3^; Andrey Kozachenko^1^; Konstantin Chingin^2^; Huanwen Chen^2*^; Vladimir Frankevich^1*^, Leila Adamyan^1^, Gennady Sukhikh^1^

^1^ *V.I. Kulakov Research Center for Obstetrics, Gynecology and Perinatology, Department of System Biology in Reproduction, 4 Oparina str., 117997 Moscow, Russia*

^2^ *Jiangxi Key Laboratory for* *Mass Spectrometry and Instrumentation, East China University of Technology, 418 Guanglan road, 330013 Nanchang, China*

*^3^ Moscow Institute of Physics and Technology, Moscow, Russia*

Corresponding authors: Dr. Vladimir Frankevich, E-mail: vfrankevich@gmail.com

Prof. Huanwen Chen, E-mail: chw8868@gmail.com

**Keywords:** tissue spray; mass spectrometry; ambient mass spectrometry; lipidomics; endometriosis.

**Tables: 4, Figures: 3**

**Table S-1.** Main patient characteristics data.

| **Age category** | **Patients, n=30** | **Frequency %** |
| --- | --- | --- |
| >26 years | 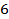 | 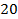 |
| 26–29.9 years | 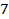 | 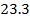 |
| 30–35.9 years | 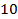 | 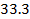 |
| 36–40.9 years | 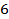 | 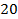 |
| >41 years | 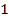 | 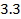 |
| **Menstrual phase** | | |
| Proliferative | 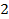 | 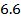 |
| Late prol./early sec. | 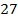 | 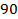 |
| Secretory | 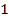 | 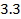 |
| Not determined | 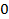 | 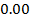 |
| **Medication use in the 6 months before** | | |
| No | 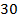 | 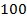 |
| Yes | 0 | 0.00 |
| **Concomitant diseases** | | |
| Adenomyosis | 6 | 20 |
| Myoma uteri | 4 | 13.3 |
| None | 20 | 66.6 |
| **BMI category** | | |
| Underweigh <18.5 | 2 | 6.6 |
| Normal 18.6–24.9 | 25 | 83.3 |
| Overweight 25-29.9 | 2 | 6.6 |
| Obese >30 | 1 | 3.3 |
| **Ethnicity** | | |
| Slovene | 27 | 90 |
| Slovene-foreign | 2 | 6.6 |
| Foreign | 1 | 3.3 |
| **The accomplishment of reproductivefunction** | | |
| Infertility | 13 | 43.3 |
| Secondary Infertility | 2 | 6.6 |
| Having children without using IVF | 8 | 26.6 |
| Having children through IVF | 0 | 0.00 |
| Not trying to conceive | 5 | 16.6 |
| Suffering from miscarriage | 5 | 16.6 |
| **Recurrence of endometriosis** | | |
| Operated for  endometriosis previously | 8 | 26.6 |
| The first incident. not operated | 22 | 73.3 |

**Table S-2.** Lipids identified in endometriotic tissue extract by HILIC-LC/MS. *m/z*’s for [M+H]^+^ ions are shown.

| **Lipid** | **Theoretical** | **Experimen- tal** | **Mass accuracy [ppm]** | **Retention time [min]** |
| --- | --- | --- | --- | --- |
| **Phosphatidylcholines** | | | | |
| PC 30:0 | 728.5200 | 728.5184 | 2 | 41.0 |
| PC O-32:0 | 742.5721 | 742.5661 | 8 | 40.8 |
| PC 32:1 | 754.5357 | 754.5326 | 4 | 40.4 |
| PC 32:0 | 756.5513 | 756.5488 | 3 | 40.5 |
| PC O-34:1 | 768.5877 | 768.5859 | 2 | 39.2 |
| PC 34:2 | 780.5513 | 780.5484 | 4 | 39.9 |
| PC 34:1 | 782.5670 | 782.5668 | 0 | 38.9 |
| PC 36:4 | 804.5513 | 804.5474 | 5 | 38.9 |
| PC 36:3 | 806.5670 | 806.5650 | 2 | 38.6 |
| PC 36:2 | 808.5826 | 808.5820 | 1 | 38.4 |
| PC 36:1 | 810.5983 | 810.5982 | 0 | 38.4 |
| PC O-38:5 | 816.5877 | 816.5836 | 5 | 38.8 |
| PC 38:5 | 830.5670 | 830.5631 | 5 | 38.4 |
| PC 38:4 | 832.5826 | 832.5806 | 2 | 38.4 |
| PC 38:3 | 834.5983 | 834.5948 | 4 | 38.3 |
| PC 40:7 | 854.5670 | 854.5655 | 2 | 38.1 |
| PC 40:6 | 856.5826 | 856.5797 | 3 | 38.1 |
| **Sphingomyelins** | | | | |
| SM 32:1 | 697.5254 | 697.5234 | 3 | 47.3 |
| SM 33:1 | 711.5411 | 711.5387 | 3 | 47.0 |
| SM 34:2 | 723.5411 | 723.5393 | 2 | 46.8 |
| SM 34:1 | 725.5567 | 725.5561 | 1 | 46.7 |
| SM 36:1 | 753.5880 | 753.5858 | 3 | 46.0 |
| SM 38:1 | 781.6193 | 781.6156 | 5 | 45.4 |
| SM 40:1 | 809.6506 | 809.6469 | 5 | 44.9 |
| SM 42:3 | 833.6506 | 833.6461 | 5 | 44.9 |
| SM 42:2 | 835.6663 | 835.6630 | 4 | 44.7 |
| SM 42:1 | 837.6819 | 837.6787 | 4 | 44.5 |
| **Phosphatidyletanoamines** | | | | |
| PE O-34:2 | 702.5432 | 702.5407 | 4 | 24.3 |
| PE 34:1 | 718.5381 | 718.5337 | 6 | 24.9 |
| PE O-36:5 | 724.5276 | 724.5236 | 5 | 23.5 |
| PE O-36:3 | 728.5589 | 728.5539 | 7 | 24.0 |
| PE O-36:2 | 730.5745 | 730.5710 | 5 | 23.9 |
| PE 36:4 | 740.5225 | 740.5190 | 5 | 24.2 |
| PE 36:2 | 744.5538 | 744.5507 | 4 | 24.6 |
| PE 36:1 | 746.5694 | 746.5638 | 8 | 24.6 |
| PE O-38:7 | 748.5276 | 748.5218 | 8 | 23.2 |
| PE O-38:6 | 750.5432 | 750.5381 | 7 | 23.2 |
| PE O-38:5 | 752.5589 | 752.5545 | 6 | 23.3 |
| PE 38:7 | 762.5068 | 762.5001 | 9 | 24.2 |
| PE 38:6 | 764.5225 | 764.5173 | 7 | 24.0 |
| PE 38:5 | 766.5381 | 766.5327 | 7 | 23.9 |
| PE 38:4 | 768.5538 | 768.5491 | 6 | 23.5 |
| PE O-40:6 | 778.5745 | 778.5676 | 9 | 23.2 |
| PE O-40:5 | 780.5902 | 780.5867 | 4 | 23.2 |
| PE 40:8 | 788.5225 | 788.5179 | 6 | 23.8 |
| PE 40:7 | 790.5381 | 790.5316 | 8 | 23.9 |
| PE 40:5 | 794.5694 | 794.5640 | 7 | 23.9 |
| PE 40:4 | 796.5851 | 796.5810 | 5 | 23.8 |
| PE 42:10 | 812.5225 | 812.5185 | 5 | 23.9 |
| PE 42:9 | 814.5381 | 814.5324 | 7 | 23.7 |

**Table S-3.** OPLS-DA models for positive ion tissue spray MS data of eutopic and ectopic endometrium. Numbers of components and samples are shown together with R^2^ and Q^2^ values which correspond to fractions of the data that the model can explain using the latent variables and fraction of the data predicted by the model according to the cross validation.

| **Model** | **Components** | **Number of samples** | **R^2^(cum)** | **Q^2^(cum)** |
| --- | --- | --- | --- | --- |
| **Pelvic vs Eutopic** | 1+4+0 | 60 | 0.96 | 0.80 |
| **Ovarian vs Eutopic** | 1+3+0 | 60 | 0.88 | 0.64 |
| **Pelvic and Ovarian vs Eutopic** | 1+3+0 | 90 | 0.89 | 0.66 |

**Table S-4.** OPLS-DA models for negative ion tissue spray MS data of eutopic and ectopic endometrium. Numbers of components and samples are shown together with R^2^ and Q^2^ values which correspond to fractions of the data that the model can explain using the latent variables and fraction of the data predicted by the model according to the cross validation.

| **Model** | **Components** | **Number of samples** | **R^2^(cum)** | **Q^2^(cum)** |
| --- | --- | --- | --- | --- |
| **Pelvic vs Eutopic** | 1+3+0 | 60 | 0.82 | 0.51 |
| **Ovarian vs Eutopic** | 1+1+0 | 60 | 0.50 | 0.30 |
| **Pelvic and Ovarian vs Eutopic** | 1+2+0 | 90 | 0.66 | 0.54 |

**Fig. S-1** Positive ion current stability check: **a** extracted ion chromatograms of some selected peaks annotated in b; **b** positive ion tissue spray mass spectra of endometriotic ovarian cyst. Relative standard deviation of meaningful signals is around 5%. Colors of lines in a correspond to colors of annotation in b.


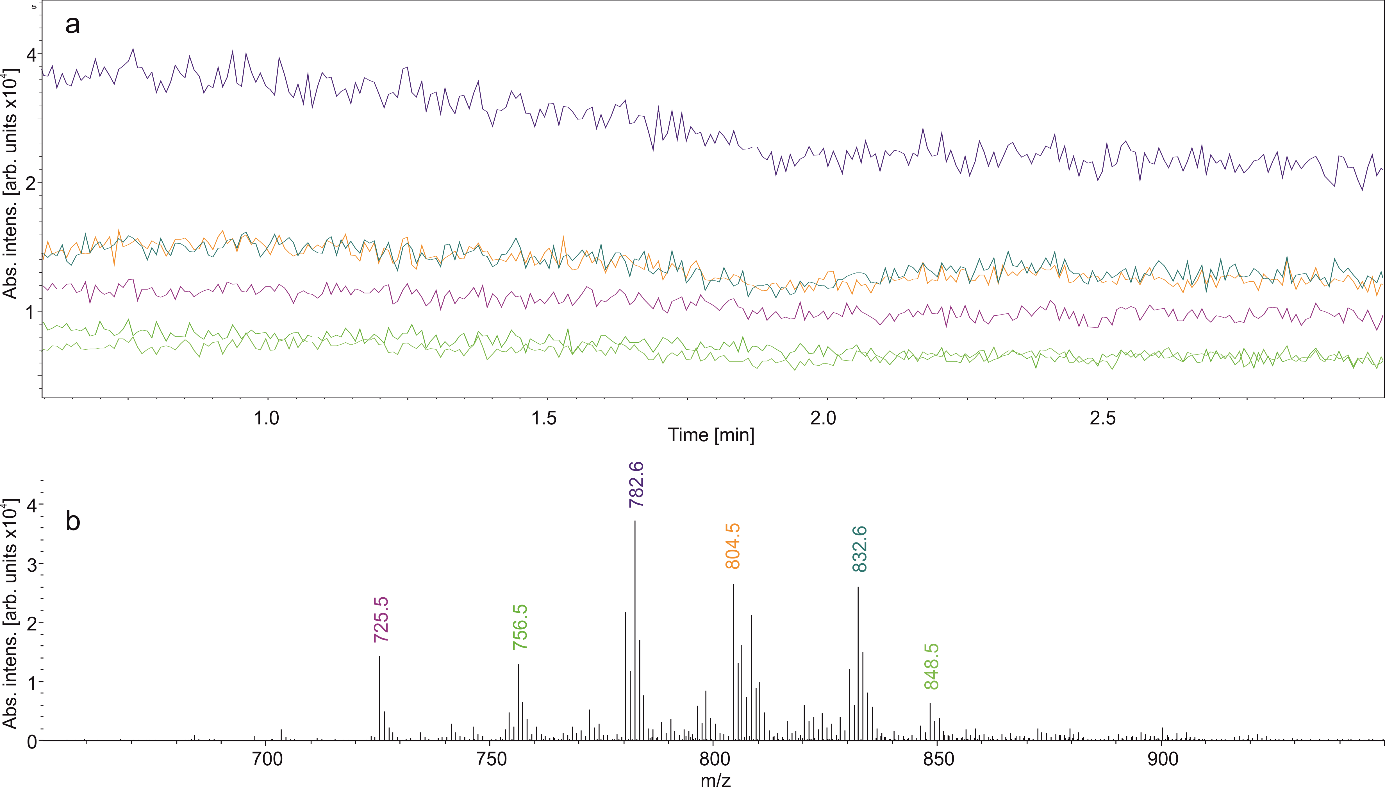


**Fig. S-2** Positive ion tandem mass spectra of peaks under identification


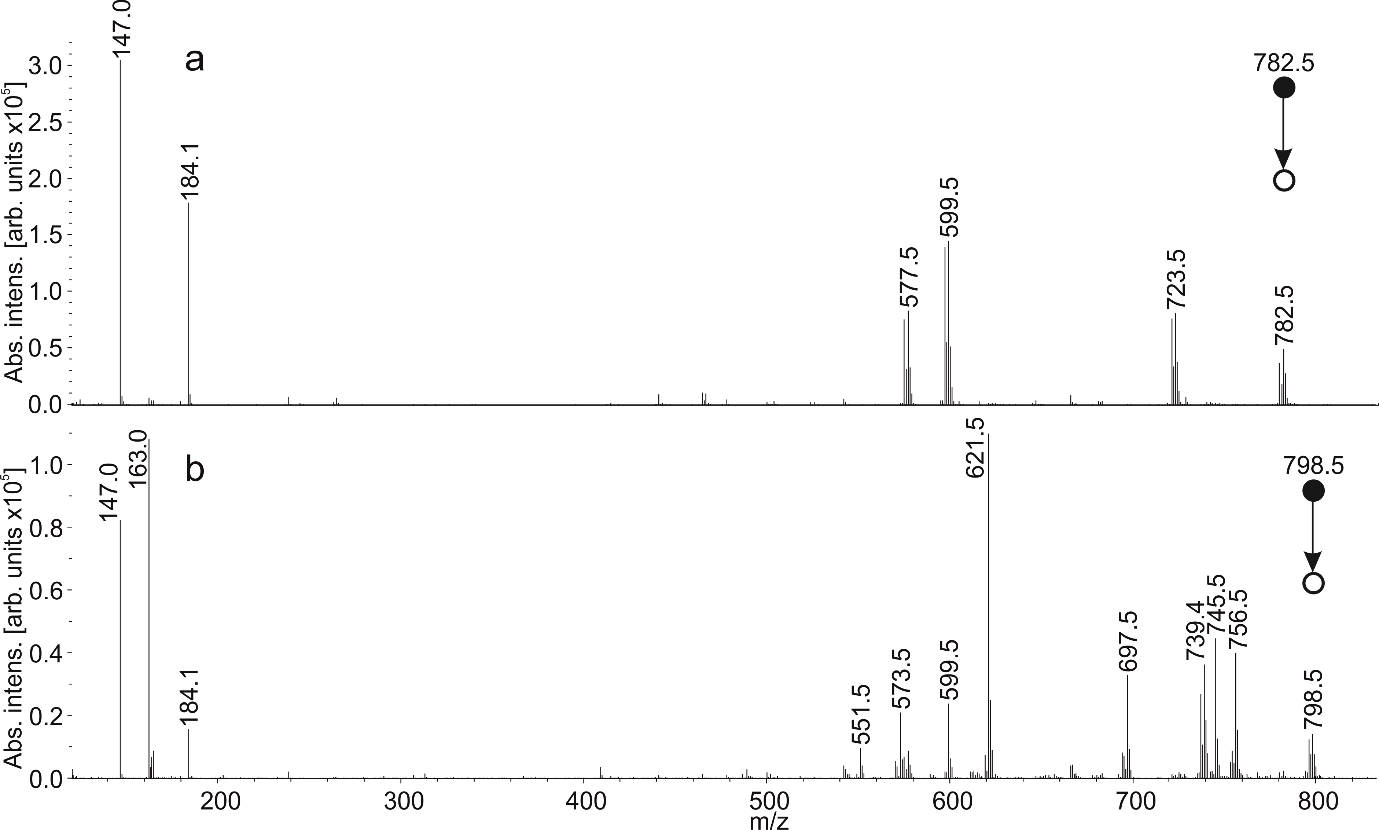


**Fig. S-3** Positive ion TIC of HILIC-LC/MS analysis of lipid extract from endometriotic tissue.


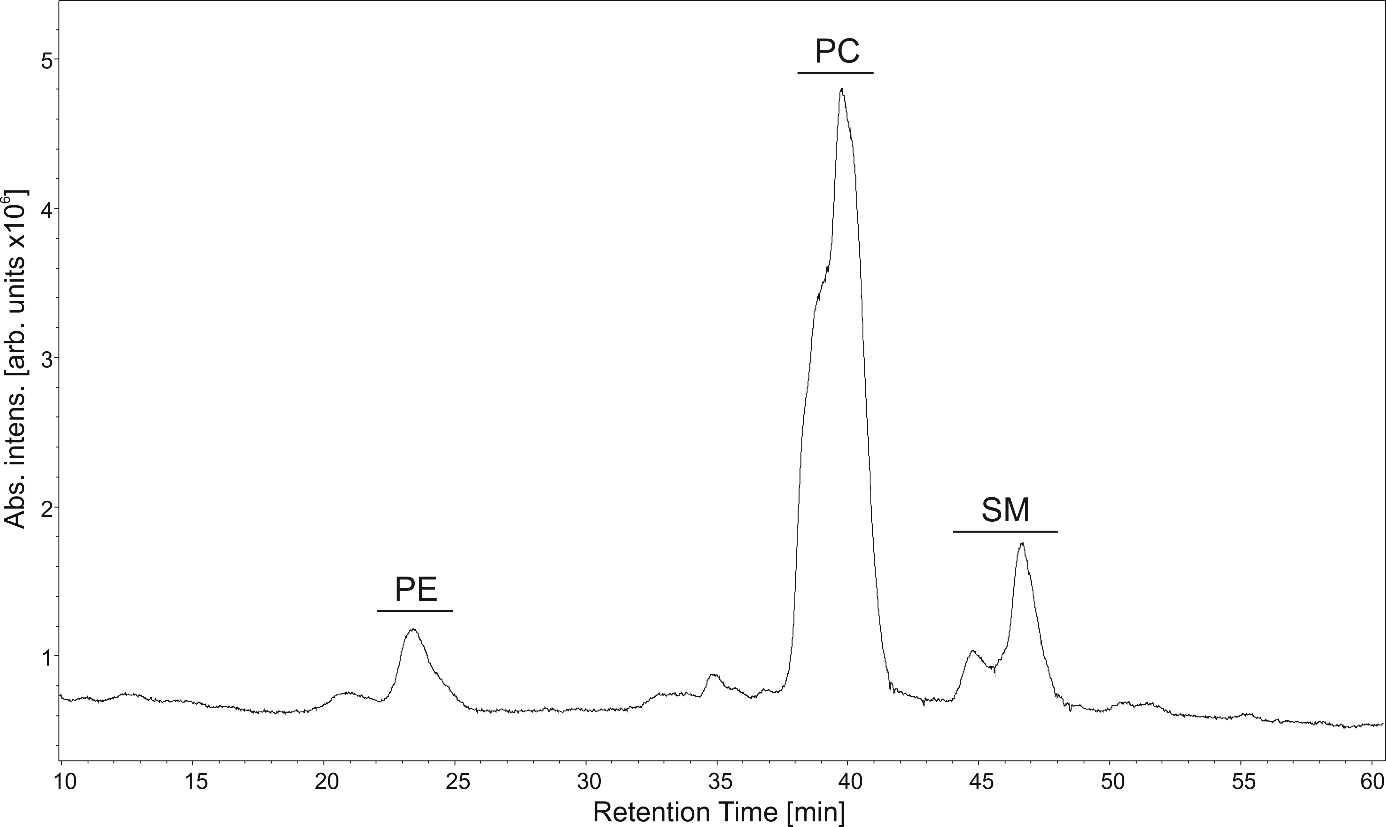

Supplement: Supplementary file 1 — Supplementary Information [file 41598_2017_2708_MOESM1_ESM.doc]
